# Cardiorespiratory Information Dynamics during Mental Arithmetic and Sustained Attention

**DOI:** 10.1371/journal.pone.0129112

**Published:** 2015-06-04

**Authors:** Devy Widjaja, Alessandro Montalto, Elke Vlemincx, Daniele Marinazzo, Sabine Van Huffel, Luca Faes

**Affiliations:** 1 Department of Electrical Engineering (ESAT)—STADIUS, KU Leuven, Leuven, Belgium; 2 Medical Information Technologies Department, iMinds, Leuven, Belgium; 3 Department of Data Analysis, Ghent University, Ghent, Belgium; 4 Faculty of Psychology and Educational Sciences, Health Psychology, KU Leuven, Leuven, Belgium; 5 IRCS-FBK and BIOtech, Department of Industrial Engineering, University of Trento, Trento, Italy; University of Adelaide, AUSTRALIA

## Abstract

An analysis of cardiorespiratory dynamics during mental arithmetic, which induces stress, and sustained attention was conducted using information theory. The information storage and internal information of heart rate variability (HRV) were determined respectively as the self-entropy of the tachogram, and the self-entropy of the tachogram conditioned to the knowledge of respiration. The information transfer and cross information from respiration to HRV were assessed as the transfer and cross-entropy, both measures of cardiorespiratory coupling. These information-theoretic measures identified significant nonlinearities in the cardiorespiratory time series. Additionally, it was shown that, although mental stress is related to a reduction in vagal activity, no difference in cardiorespiratory coupling was found when several mental states (rest, mental stress, sustained attention) are compared. However, the self-entropy of HRV conditioned to respiration was very informative to study the predictability of RR interval series during mental tasks, and showed higher predictability during mental arithmetic compared to sustained attention or rest.

## Introduction

Work-related stress is estimated to cost the US industry yearly over 300 billion USD due to lower productivity, absenteeism, turnover and medical, legal and insurance costs [1, 2]. In Europe, it has been estimated that work-related depression, a possible outcome of prolonged exposure to stress, costs 617 billion EUR annually [3]. Additionally, job stress has been identified as an important risk factor for other mental health problems, musculoskeletal disorders and cardiovascular diseases [4–6].

When experiencing stress, the fight-or-flight response is activated to enable us to quickly respond to life-threatening situations. This, in turn, stimulates the sympathetic branch of the autonomic nervous system (ANS), acting amongst others on the cardiovascular system [7]. The autonomic control on the cardiovascular system is often studied by means of heart rate variability (HRV), which describes the interaction between the sympathetic and vagal nervous system [8]. Because HRV can easily be assessed by means of simple ECG recordings, it is a popular tool that has also often been used to study the response of the cardiovascular system to mental stress; typically, the heart rate increases and the sympathovagal balance shifts towards sympathetic dominance, while the vagal modulation is strongly reduced [5, 9–13].

Not only the heart rate, but also the breathing frequency increases due to exposure to mental stress [14–16]. Vlemincx et al. also reported the effect on respiratory variability, and found a reduction in total respiratory variability during sustained nonstressful attention while the opposite occurred during mental load [16]. Moreover, it is also important to note that respiration has a major influence on HRV. This phenomenon is called respiratory sinus arrhythmia (RSA), and comprises the modulation of the heart rate with respiration. Though RSA has been identified as a vagally-mediated phenomenon [17–19], this has been debated since researchers found that RSA magnitude and vagal activity can dissociate depending on the respiratory frequency and tidal volume [20–23]. Therefore, a combined cardiorespiratory analysis during mental stress is needed. Previous combined studies reported that mental stress decreases RSA [24] and reduces the cardiorespiratory synchronization epochs [25]. In the past years, we have tried to take the influence of respiration into account by separating respiratory-related heart rate variations from nonrespiratory-related variations; in [26], we have conducted time-frequency analyses during mental stress testing, in which respiration was taken into account by computation of partial time-frequency spectra. In another study, we have shown that by separating respiratory influences from nonrespiratory-related heart rate variations, an almost perfect classification in periods of rest and mental stress can be obtained [27].

In this study, we aim to continue with this approach of a combined analysis of cardiorespiratory time series during mental stress testing. Now, we will use information dynamics to assess information storage and internal information of HRV, and information transfer and cross information from respiration to HRV [28]. Information theory has proven to be useful to assess directional interactions between cardiorespiratory time series [28–30], and we hypothesize that information-theoretic measures may reveal altered cardiorespiratory patterns during mental stress. In this paper, measures of information storage and transfer are estimated via a nonlinear model-free approach [31, 32]. Additionally, not only the response to mental arithmetic is evaluated, also the response to a nonstressful attention task is assessed.

## Information Decomposition

Consider a system that consists of two interacting subsystems *X* and *Y*, and we are interested in the information contained in the present sample *Y*_*n*_. Let $V_{n}^{X}=[X_{n-1},X_{n-2},\ldots ]$ and $V_{n}^{Y}=[Y_{n-1},Y_{n-2},\ldots ]$ be the past states of *X* and *Y* respectively, then we can define the predictive information of our target process *Y* as:

$$\begin{array}{c} P_{Y}=H\left(Y_{n}\right)-H\left(Y_{n}|V_{n}^{X,Y}\right), \end{array}$$ (1)

where *H*(*Y*_*n*_) is the Shannon entropy that is given by *H*(*Y*_*n*_) = −∑*p*(*y*_*n*_)ln *p*(*y*_*n*_), and where $H(Y_{n}|V_{n}^{X,Y})$ is the conditional entropy with $V_{n}^{X,Y}=[V_{n}^{X},V_{n}^{Y}]$ the past states of both *X* and *Y*. The predictive information *P*_*Y*_, then, determines how much of the information carried by *Y*_*n*_ can be predicted by the knowledge of the past of *X* and *Y*.

Let *X*, the driver signal, be the respiratory signal and *Y*, the target signal, the RR interval series, then we can decompose the predictive information *P*_*Y*_ in one term that describes the information transfer going from the respiratory signal *X* to the RR interval series *Y*, and another term that contains the information storage of *Y*:

$$\begin{array}{c} P_{Y}=\underset{S_{Y}}{\underset{︸}{H\left(Y_{n}\right)-H\left(Y_{n}|V_{n}^{Y}\right)}}+\underset{T_{X\to Y}}{\underset{︸}{H\left(Y_{n}|V_{n}^{Y}\right)-H\left(Y_{n}|V_{n}^{X,Y}\right)}}, \end{array}$$ (2)

with *S*_*Y*_ the self-entropy, a measure of information storage that quantifies how much information carried by *Y*_*n*_ can be predicted by the knowledge of its own past [33], and *T*_*X* → *Y*_ the well-known transfer entropy, a measure of information transfer that quantifies how much of the information carried by *Y*_*n*_ can be predicted by the past of *X*, conditioned to the knowledge of the past of *Y* [34]. As such, *T*_*X* → *Y*_ describes the additional predictability that the past of *X* brings about the present of *Y* that was not brought already by the past of *Y*.

Alternatively, the predictive information of *Y* can be decomposed by first considering the past of driver *X*, leading to

$$\begin{array}{c} P_{Y}=\underset{C_{X\to Y}}{\underset{︸}{H\left(Y_{n}\right)-H\left(Y_{n}|V_{n}^{X}\right)}}+\underset{S_{Y|X}}{\underset{︸}{H\left(Y_{n}|V_{n}^{X}\right)-H\left(Y_{n}|V_{n}^{X,Y}\right)}}, \end{array}$$ (3)

with *C*_*X* → *Y*_ the cross-entropy from *X* to *Y* and *S*_*Y*|*X*_ the conditional self-entropy [28]. *C*_*X* → *Y*_ is a measure of cross information that quantifies how much information carried by *Y*_*n*_ can be predicted by the knowledge of the past of *X*. *S*_*Y*|*X*_ constitutes a measure of internal information that describes how much information carried by *Y*_*n*_ can be predicted by its own past, conditioned to the past of *X*, and thus *S*_*Y*|*X*_ describes the additional predictability that the past of *Y* brings about its present that was not brought already by the past of *X*.

In [28], an extensive theoretical analysis of both decompositions of predictive information was carried out. It was shown that self-entropy measures the information storage, but cannot be related in a straightforward way to the internal dynamics of the target process *Y*, as the past of *Y* may be driven by the past of *X*. On the other hand, the conditional self-entropy reflects the internal information in the target process because it will always be zero in the absence of internal dynamics in the target, and it is not influenced by the dynamics of the driver process. Likewise, the cross-entropy measures the cross information from the driver process *X* to the target process *Y*, and thus, it reflects the information that is carried by the target process that can be explained by the driver’s past, regardless of the origin of the driver’s past. Therefore, the cross-entropy cannot be taken as an index of causality since it can be nonzero, even in the absence of any causal link from driver to target. Transfer entropy, on the other hand, measures the information transfer from the driver to the target process and will always be zero in the absence of a causal link from driver to target.

With *X* = *RSP* the respiratory signal and *Y* = *RR* the RR interval series, we then have *P*_*RR*_, *T*_*RSP* → *RR*_, *S*_*RR*_, *C*_*RSP* → *RR*_ and *S*_*RR*|*RSP*_ as information-theoretic measures, where *T*_*RSP* → *RR*_ and *C*_*RSP* → *RR*_ can be considered as indices reflecting the cardiorespiratory dynamics.

The entropies and conditional entropies appearing in Eqs (1–3) are estimated using a nonlinear model-free approach that is able to capture in principle any type of (linear and nonlinear) dynamics underlying the observed interactions. Additionally, a test is set up to determine the significance of the contribution of nonlinear dynamics with respect to linear interactions.

### Estimation of Information Dynamics

In the model-free estimation approach adopted in this study [31, 32], the conditional entropy is computed according to a procedure for nonuniform conditioning, designed to select—among all possible lagged components forming the past of *X* and *Y*—only those components which contribute significantly to the description of the present of the target *Y*_*n*_. This is essential to counteract the curse of dimensionality and provide reliable estimates of information dynamics in short data sets [31, 32, 35]. In essence, the procedure for nonuniform conditioning builds iteratively the vector $V_{n}^{X,Y}$ selecting terms from a set of candidate components Ω = {*X*_*n*−1_, …, *X*_*n*−*L*_, *Y*_*n*−1_, …, *Y*_*n*−*L*_}. Starting from an empty vector $V_{n}^{X,Y}=[\cdot ]$, the procedure tests at each step all the candidate components of Ω, computing for each candidate the mutual information between the set of components selected up to that step, $V_{n}^{X,Y}$, incremented with the test candidate, and the present of the target; the candidate leading to the maximum mutual information is selected and included in $V_{n}^{X,Y}$. This achieves a criterion for maximum relevance and minimum redundancy in the selection of components. Finally, the selection is terminated by means of a randomization test which generates surrogates of the selected component and uses them to set a significance threshold for the mutual information. This allows to include in the final conditioning vector $V_{n}^{X,Y}$ only the components that bring statistically significant information to the present of the target. After termination of the procedure, the subvectors $V_{n}^{X}$ and $V_{n}^{Y}$ are deduced from $V_{n}^{X,Y}$ by simply taking the terms that belong respectively to the past of *X* and the past of *Y*. Finally, the information measures *P*_*Y*_, *S*_*Y*_, *S*_*Y*|*X*_, *C*_*X* → *Y*_, and *T*_*X* → *Y*_ are estimated from the embedding vectors $V_{n}^{X,Y}$, $V_{n}^{X}$ and $V_{n}^{Y}$ as indicated in Eqs (1–3). The computation of all conditional entropy and mutual information terms is based on coarse-grained quantization using *Q* quantization levels, followed by approximation of the probability distributions with the frequencies of occurrence of the quantized values. In our study, lags up to 5 *s* (*L* = 10) were considered to cover the past of the respiratory series *X* and the RR interval series *Y*, and *Q* = 8 quantization levels were adopted for entropy estimation.

All entropies are computed using the MuTE toolbox [32].

### Testing Significance and Nonlinearity

The computation of the measures of information dynamics above described is complemented with statistical tests aimed at assessing the significance of each measure, and the contributions of nonlinear dynamics to the measure.

The information about the statistical significance is extracted from the randomization test employed by the procedure for candidate selection. Indeed, as a result of the selection procedure and the exploitation of the full and reduced vectors $V_{n}^{X,Y}$, $V_{n}^{X}$ and $V_{n}^{Y}$, the estimated information measures *P*_*Y*_, *S*_*Y*_, *S*_*Y*|*X*_, *C*_*X* → *Y*_, and *T*_*X* → *Y*_ result as strictly positive when at least one relevant candidate component is selected from the past of *X* or *Y* according to the definitions in Eqs (1–3), thus yielding statistically significant predictive information, information storage, internal information, cross information or information transfer, and are exactly zero otherwise. Therefore, we considered as statistically significant the information measures when they are larger than zero, and nonsignificant when they are exactly zero.

Further, in order to determine the significance of the contribution of nonlinear dynamics on the predictive information and on the measures of its decomposition, a test was set up to estimate the information dynamics of ‘linearized’ versions of each observed bivariate time series. For this purpose, firstly, 100 linearized surrogates of the cardiorespiratory time series are constructed for each bivariate time series. These surrogates are created from a vector autoregressive (VAR) model that is estimated on the bivariate process [36], and is then fed with independent realizations of pairs of white uncorrelated Gaussian noises with the same variance as the residuals of the estimated VAR model. This yields several realizations of linear Gaussian processes sharing the same linear structure of the original observed bivariate process. The optimal model order is determined via the minimum description length method. Next, *P*_*Y*_, *S*_*Y*_, *S*_*Y*|*X*_, *C*_*X* → *Y*_, and *T*_*X* → *Y*_ of the 100 surrogates are computed using the model-free approach, giving rise to the distribution of these measures under the null hypothesis of linear Gaussian cardiorespiratory time series. The null hypothesis is rejected when the original value obtained using the model-free approach falls outside the 95% confidence interval of the null distribution, thus indicating the presence of nonlinear dynamics.

## Data and Statistical Analysis

### Data Acquisition

The data were recorded at the Faculty of Psychology and Educational Sciences of the KU Leuven (Leuven, Belgium) [11, 16]. The electrocardiogram (sampling frequency *f*_*s*_ = 200 Hz) and respiration (*f*_*s*_ = 50 Hz) of 40 healthy students (age: 18–22 years) were simultaneously recorded using the LifeShirt System (Vivometrics Inc., Ventura, CA). The tidal volume was taken as respiratory signal and was estimated by means of inductive plethysmography around the abdomen and ribcage.

During the experimental protocol, the participants were instructed to conduct two types of tasks. The first type of task was a mental arithmetic task that was designed to induce mental stress. They indicated the correct answer of three solutions using a mouse cursor after which feedback was given. The experimenter was seated next to the participant. A free movie ticket was awarded to the five participants who achieved the most correct answers. Mental arithmetic is often used to induce stress, as it has been shown that it affects several physiological indices of stress [37, 38]. The second task was a nonstressful attention task where the participants had to indicate the largest number on a computer using a mouse cursor. The attention task required the same motor movement as the mental arithmetic task, but the task difficulty was extremely low; there were no time constraints, nor performance rewards.

The whole protocol consisted of an attention task (AT) and 2 mental stress tasks (MT1 and MT2), each followed by a recovery period. The order of the tasks was randomized. Prior to any task, a baseline recording (BASE) was taken during which the participants watched a relaxing documentary. All periods had a duration of 6 minutes. Prior to the experiment, the participants were instructed not to speak or move their lips, and not to change posture or move their body except for the dominant hand to use the mouse cursor.

All participants provided written informed consent. The experiment was approved by the Ethics Committees of the Department of Psychology and Educational Sciences and of the Faculty of Medical Sciences of the KU Leuven. The study was in accordance with the Declaration of Helsinki (2008).

### Data Preprocessing

The R peaks in the ECG are detected using the Pan-Tompkins algorithm. Parabolic interpolation using the 5 samples around the detected R peak is conducted to obtain an accuracy of 1 ms when composing the tachogram. Next, the tachogram and respiratory signal are resampled at 2 Hz using cubic spline interpolation. Baseline wander of the respiratory signal is removed using a high-pass filter with a cut-off frequency of 0.05 Hz, while the tachogram is detrended according to [39]. In order to reduce the transient behaviour, present mainly in the first minute of each task [26], and obtain stationary conditions, only the last 5 minutes of each period are selected for the analysis of information dynamics. Possible delays between the respiratory drive and the recorded tidal volume that might affect the driver-response relation, are taken into account by including a time lag of 0.5 *s* in the respiratory signal [40]. In order to obtain a more robust estimation using the binning approach, ordinal sampling of the cardiorespiratory time series was conducted, thereby transforming the signal into its ranks, as is done in most non-parametric statistical tests [41]. Next, stationarity was tested according to the test described in [42]. Signals that failed the stationarity test are discarded from the analysis.

All processing steps of the data are conducted in MATLAB R2012a (MathWorks, Natick, MA).

### Statistical Analysis

After computation of *P*_*RR*_, *T*_*RSP* → *RR*_, *S*_*RR*_, *C*_*RSP* → *RR*_ and *S*_*RR*|*RSP*_, differences in cardiorespiratory information dynamics between the resting, attention, and two stress conditions are assessed by means of the Friedman test. Tukey’s honestly significant difference criterion is used to take multiple comparisons into account. A significance level of *α* = 0.05 is used.

## Results

**Fig 1:**
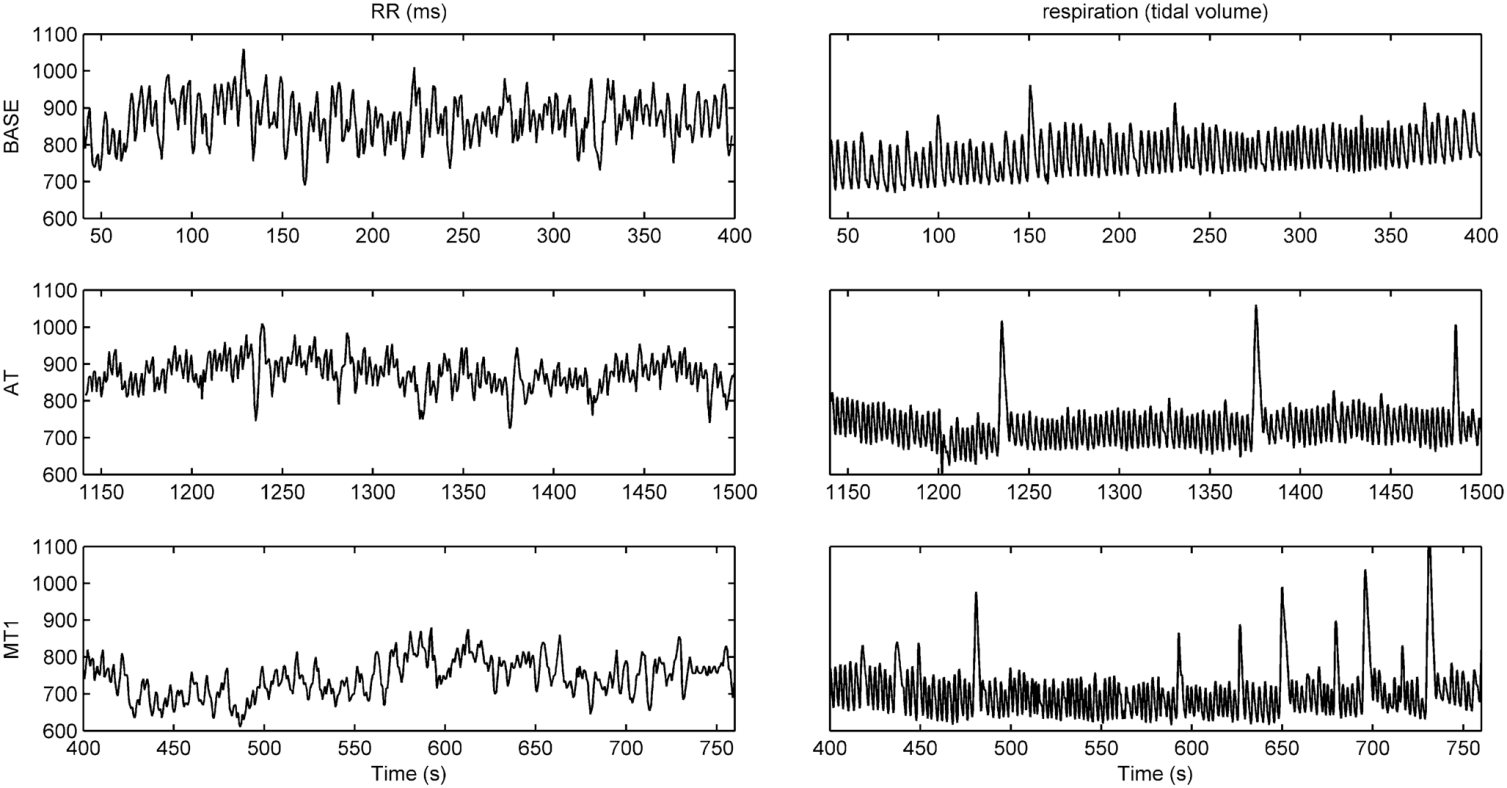
RR interval series and respiratory signals during baseline (BASE), sustained attention (AT) and the first mental stress task (MT1).

Fig 1 displays the RR interval series and the respiratory signals during the baseline recording, the attention and the first stress task. From these plots, it is immediately clear that mental arithmetic increases both the heart rate and the respiratory rate. During the attention task, there is also an increase in both rates, but not as much as during MT1. Both during AT and MT1, HRV decreases significantly compared to BASE, though there is nearly any difference between both tasks. An elaborate HRV study with appropriate statistical analysis on these data is presented in [11].

For the study of information dynamics, data of 5 subjects were discarded due to the lack of stationarity.

### Significance and Nonlinearity

**Fig 2:**
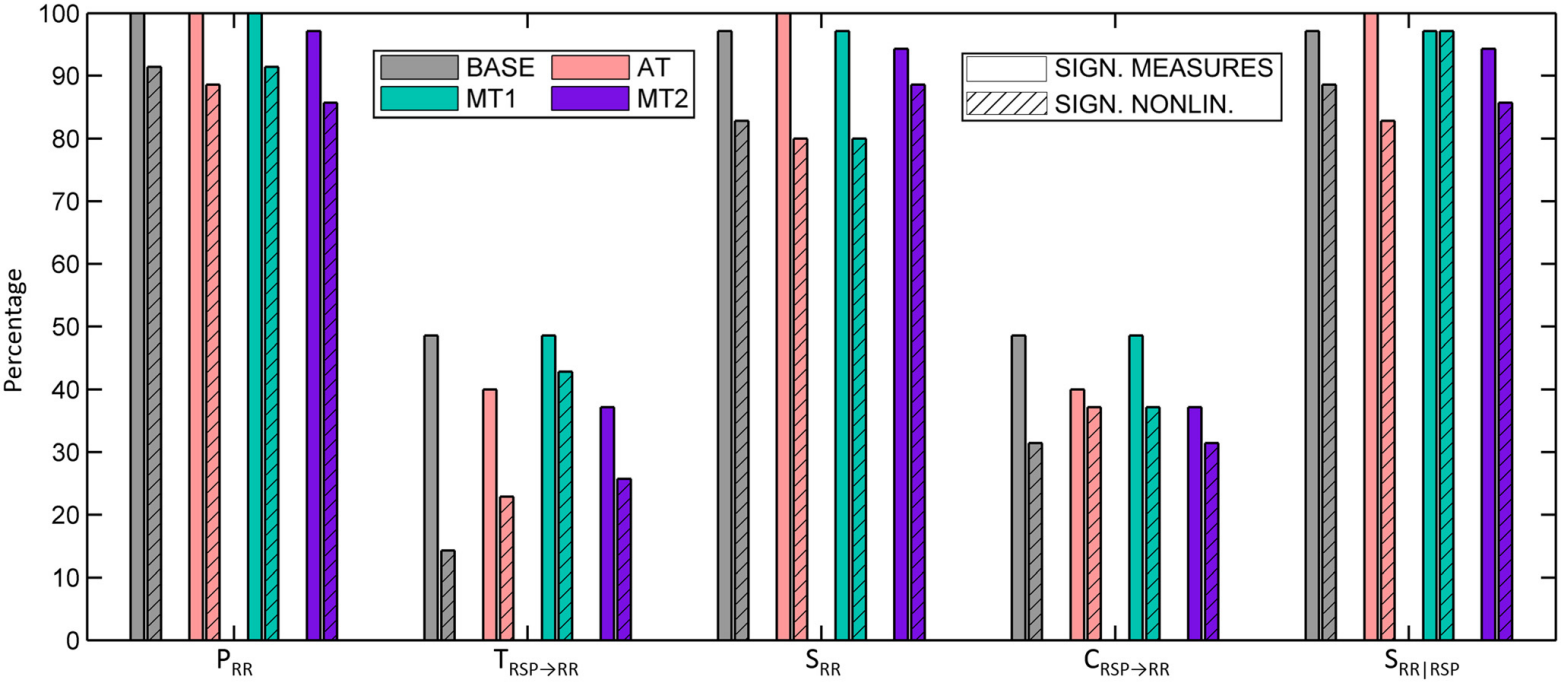
Bar graphs indicating the percentage of significant information-theoretic measures (plain) and the percentage of significant nonlinearities (hatched), during baseline (BASE), sustained attention (AT) and the mental stress tasks (MT1 and MT2).

Fig 2 shows the percentage of subjects that have strictly positive information-theoretic measures (given in plain colored bars). While for *P*_*RR*_, *S*_*RR*_ and *S*_*RR*|*RSP*_, almost all subjects have strictly positive values, not even half of the subjects have any information transfer or cross information, indicating that in the conditioning vector, in many subjects, no candidate of the driver process was selected.

The procedure to test the presence of nonlinearities was followed for each information-theoretic measure, each subject and each mental state. The percentage of rejected null hypotheses is given in the hatched colored bars in Fig 2, indicating that for *P*_*R*_, *S*_*RR*_ and *S*_*RR*|*RSP*_, in the majority of the subjects and in all mental states, the null hypothesis was rejected, and thus significant nonlinearities were observed. In fact, during MT1, the null hypothesis for *S*_*RR*|*RSP*_ was rejected in all subjects except for the one which also did not have a significant *S*_*RR*|*RSP*_. For *T*_*RSP* → *RR*_ and *C*_*RSP* → *RR*_, the null hypothesis was rejected in not even half of the subjects, which is evident, given the percentage of significant transfer and cross-entropies. Interestingly, nonlinearities contributed to the transfer entropy *T*_*RSP* → *RR*_ only in a small subset of the significant measures in the baseline condition, and in almost all cases during MT1.

### Information Dynamics

**Fig 3:**
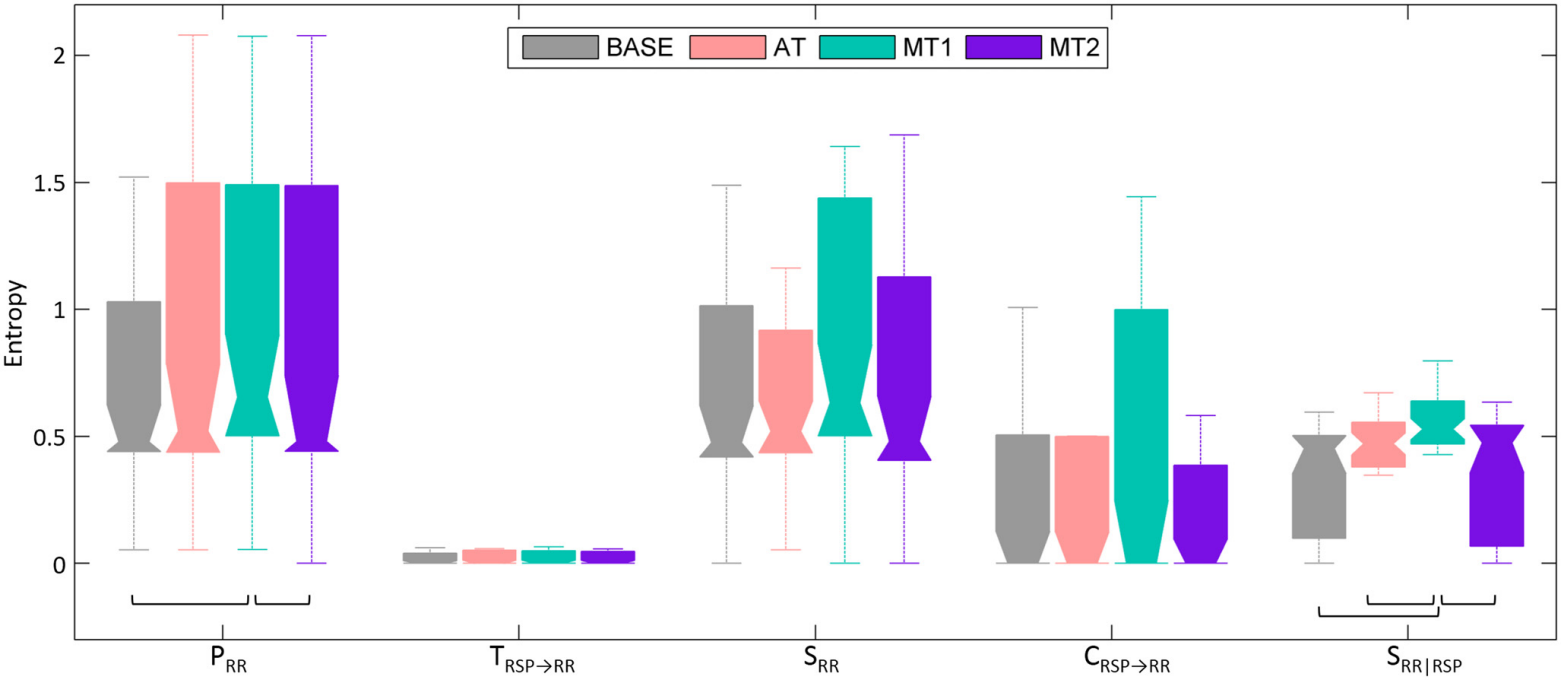
Boxplots of information-theoretic measures *P*_*RR*_ (*p* < 0.01), *T*_*RSP* → *RR*_ (*p* > 0.05), *S*_*RR*_ (*p* > 0.05), *C*_*RSP* → *RR*_ (*p* > 0.05) and *S*_*RR*|*RSP*_ (*p* < 0.0001), estimated using the nonlinear model-free approach, during baseline (BASE), sustained attention (AT) and the mental stress tasks (MT1 and MT2). The square brackets indicate significant differences between those two mental states as determined by the Friedman test and post hoc contrasts.

Fig 3 displays boxplots of *P*_*RR*_, *T*_*RSP* → *RR*_, *S*_*RR*_, *C*_*RSP* → *RR*_ and *S*_*RR*|*RSP*_ during BASE, AT, MT1 and MT2, estimated using the nonlinear model-free approach. The post hoc contrasts reveal that BASE and MT2 are significantly different from MT1 in terms of *P*_*RR*_ (*p* < 0.01). *T*_*RSP* → *RR*_, *S*_*RR*_ and *C*_*RSP* → *RR*_ present no differences between mental states, while *S*_*RR*|*RSP*_ is significantly larger during the first mental task than during the other states (*p* < 0.0001).

**Fig 4:**
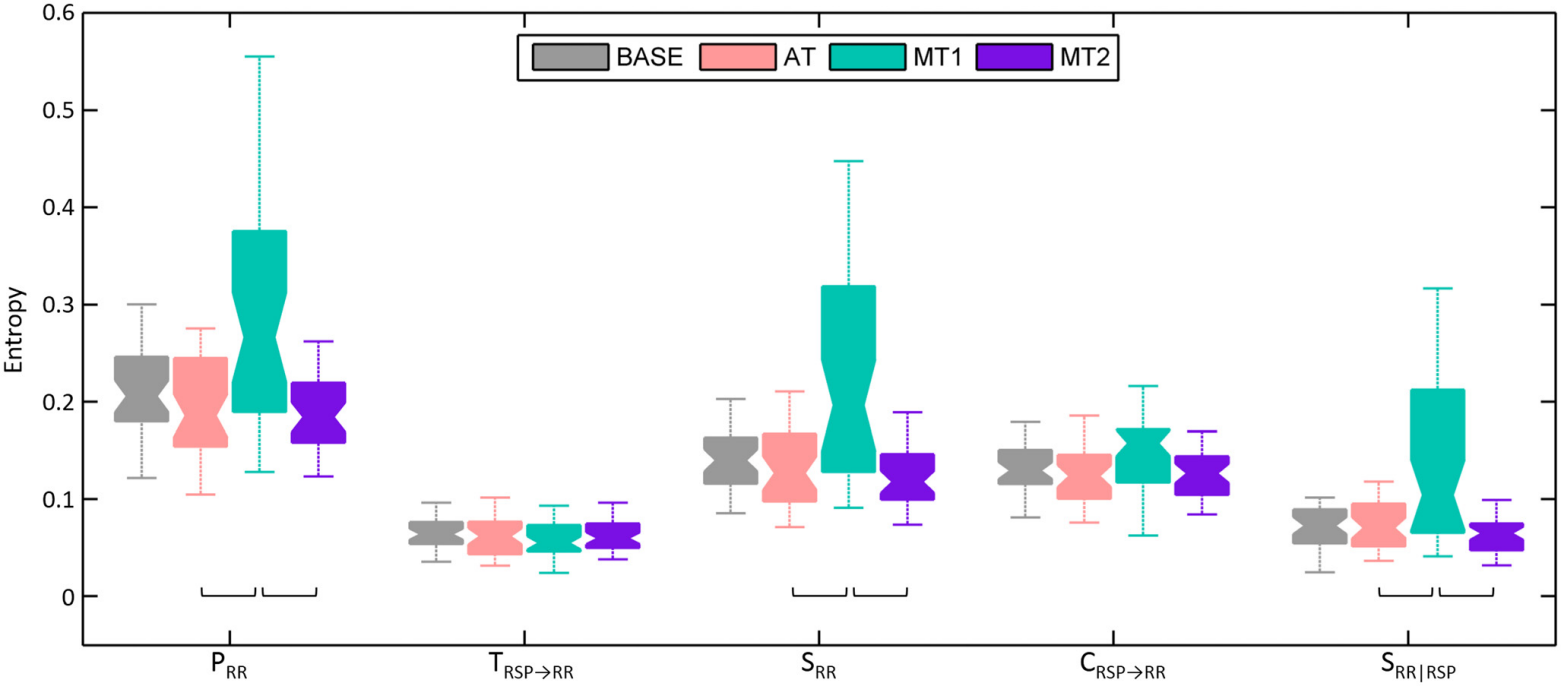
Boxplots of mean information-theoretic measures *P*_*RR*_ (*p* < 0.01), *T*_*RSP* → *RR*_ (*p* > 0.05), *S*_*RR*_ (*p* < 0.001), *C*_*RSP* → *RR*_ (*p* > 0.05) and *S*_*RR*|*RSP*_ (*p* < 0.01), estimated using the model-free approach using the linear surrogates as input, during baseline (BASE), sustained attention (AT) and the mental stress tasks (MT1 and MT2). The square brackets indicate significant differences between those two mental states as determined by the Friedman test and post hoc contrasts.

In order to determine the importance of nonlinear cardiorespiratory dynamics, boxplots of *P*_*RR*_, *T*_*RSP* → *RR*_, *S*_*RR*_, *C*_*RSP* → *RR*_ and *S*_*RR*|*RSP*_ for the linear surrogates are displayed in Fig 4. For each subject and each mental state, the mean of the 100 surrogates was taken as input for the boxplots. Comparing these boxplots to the ones of Fig 3 obtained using the real cardiorespiratory time series and the model-free approach, several differences can be observed; *P*_*RR*_ and *S*_*RR*_ differ significantly between AT and MT1, whereas when nonlinear interactions are taken into account, no differences between those two states for these measures could be observed. Additionally, *S*_*RR*_ is also significantly different between MT1 and MT2, while for *S*_*RR*|*RSP*_ there is no difference between BASE and MT1 when the linear surrogates are used.

## Discussion

### Mental Arithmetic and Sustained Attention

In a previous study using these data, traditional time and frequency domain HRV measures were computed to study the effect of mental arithmetic and sustained attention [11]. Almost all measures showed significant differences between BASE and the tasks, with increased heart and respiratory rates, and reduced HRV during the tasks. The strongest effect was observed during MT1, then during AT. A habituation effect was noted in the HRV measures during the second mental task. Between the tasks, differences were found between MT2 and the other tasks, but only few measures were able to prove the additional mental load of MT1 compared to AT, as could also be visually observed in Fig 1. Thus, in terms of discrimination of different mental states, no new differences could be found with the analysis of information dynamics that could not be observed with the traditional HRV analysis. The added value lies in the type of information that we can acquire using information dynamics. With the results of these analyses, we can gain more insight in the complex, underlying behavior of the cardiorespiratory system during these different mental states in terms of entropy, and thus predictability of the RR interval time series.

From Fig 3, we could only observe differences between mental states for *P*_*RR*_ and *S*_*RR*|*RSP*_. Though transfer *T*_*RSP* → *RR*_ and cross-entropy *C*_*RSP* → *RR*_ are both measures of cardiorespiratory coupling and were expected to decrease during mental stress, we could not witness any task effect. It is, however, important to note that RSA is in essence a measure of gain of the cardiorespiratory interaction and not of cardiorespiratory coupling; one could have differences in gain, but with a preserved coupling. In this analysis, we already found a low coupling during rest, which stayed low during other mental states. This suggests that the reduction in RSA amplitude during mental stress, as found in [11], is not accompanied by a reduction in cardiorespiratory coupling. Nevertheless, our results document an increased contribution of nonlinear dynamics to the transfer entropy *T*_*RSP* → *RR*_ during MT1 compared to rest (Fig 2), thus suggesting that mental stress may induce an alteration in the nature of cardiorespiratory interactions, although this alteration seems not to have an impact on the magnitude of the coupling.

We also observed in Fig 3 that the differences in *P*_*RR*_ emanate from the internal information as determined by *S*_*RR*|*RSP*_. This conditional self-entropy indicates how well the RR interval series can be predicted based on information of its own past, taking at first the information of respiration into account. The higher values of *S*_*RR*|*RSP*_ during MT1 indicate that the RR intervals can be better predicted than during the other states and thus reveals that the cardiac system is more predictable when experiencing mental stress. The finding of higher predictability of *RR* confirms previous findings suggesting a reduction of HRV complexity during stress [12]. Taking into account that predictability of the heart rate is typically related to an unhealthier cardiovascular system that shows a reduced rapidity to respond to bodily demands [43], the difference in conditional self-entropy is in accordance with our hypothesis. Additionally, *S*_*RR*|*RSP*_ is not only able to distinguish MT1 from a resting baseline condition, but also from a lower level of mental load, i.e. during AT. *S*_*RR*|*RSP*_ is also able to describe the habituation effect during MT2 compared to MT1, and as such seems a valuable measure to reflect different levels of sympathetic activation.

It is also interesting to note here that the predictive information already gave a hint about the changes in HRV complexity during mental stress; however, these changes are statistically more evident after accounting for respiration, i.e. by computing the conditional self-entropy rather than the predictive information. Additionally, the conditional self-entropy can also be seen as the internal memory of the tachogram, which appears to be stronger during mental stress, and thus points to reduced flexibility and reactivity of the heart. This has been related to an increase in sympathovagal balance, which is indeed expected to occur during mental stress [44].

### Information Decomposition

Two decompositions of the predictive information have been proposed, each with their own advantages and disadvantages. The information transfer as quantified by *T*_*RSP* → *RR*_ proves real transfer of information, but might underestimate it, especially when the interactions are highly unidirectional [28]. In contrast to what we hypothesized, neither *T*_*RSP* → *RR*_ nor *C*_*RSP* → *RR*_ are affected by the mental state. These measures of information transfer and cross information are indices of the cardiorespiratory coupling, but in a different way than RSA, as mentioned before. In addition, the low values for *T*_*RSP* → *RR*_ indicate that the additional predictability of the RR interval series based on *RSP* given the past of *RR* is very limited. This can be explained by the fact that in the traditional decomposition of *P*_*RR*_, we first consider the past of the target process, thereby favouring the information storage.

However, the information storage as quantified by *S*_*RR*_ might also contain some information from the driver that is captured in the past of *RR*. *S*_*RR*|*RSP*_, on the other hand, proves internal information of the RR interval series since it will be zero in the absence of internal dynamics in the target series [28]. Using the linear surrogates, no differences between *S*_*RR*_ and *S*_*RR*|*RSP*_ can be noted, apart from a constant bias. However, when nonlinear interactions are taken into account, the conditional self-entropy has more discriminative power, whereas the self-entropy has none. It is thus important for cardiorespiratory time series to condition first on the past of *RSP* to determine the information carried by *RR* that is unrelated to respiration. The obtained results confirm previous HRV studies in which the effect of respiration was taken into account by separating the tachogram into a component that is strictly related to respiration, and a component that contains all residual heart rate variations [27]. We found that the residual component of the tachogram is highly informative to classify periods of rest and mental stress, while the component related to respiration does not have discriminative power. The latter can be associated to the self-, cross- and transfer entropy while the residual component can be linked to the conditional self-entropy.

### Linear versus Nonlinear Information Dynamics

From Figs 3 and 4, it can be observed that *P*_*RR*_, *S*_*RR*_ and *S*_*RR*|*RSP*_ differ significantly when assessed on the original data and on their linear counterparts. The higher magnitudes that are observed, are in large part determined by the significant contribution of nonlinear dynamics when the real data were used (cfr. Fig 2). It is, however, also important to add that when we look at the candidates that were selected for the conditioning vector, we can notice a difference in the number of candidates that were chosen using the real cardiorespiratory time series and using the linear surrogates; on average, 2.8 candidates were selected with the real data, whereas 2.25 candidates were chosen with the linear surrogates. A higher number of candidates in the conditioning vector may create some bias towards zero in the conditional entropy, and thus some bias towards higher values in the information-theoretic measures we consider. This bias may partly explain the differences in entropy magnitudes between the real data and the linear surrogates. No differences could be noted between the number of candidates that were selected in several mental states.

When further comparing the information-theoretic measures of the real data and their linear surrogates, other differences between mental states were found for *P*_*RR*_, *S*_*RR*_ and *S*_*RR*|*RSP*_. These differences could possibly originate from a larger nonlinear predictability during MT1 and AT. In the traditional decomposition of *P*_*RR*_, the increased nonlinearities in MT1 and AT seem to be distributed over both *T*_*RSP* → *RR*_ and *S*_*RR*_, thereby abolishing the differences in mental states that were seen in *S*_*RR*_ when the linear surrogates were used. On the other hand, in the alternative decomposition of *P*_*RR*_, the nonlinear dynamics seem to contribute substantially to the internal information of *RR* during the first mental task, leading to significantly larger values during MT1 for *S*_*RR*|*RSP*_ compared to all other mental states. The increase of the impact of nonlinear dynamics on *RR* during AT seems to be distributed over *C*_*RSP* → *RR*_ and *S*_*RR*|*RSP*_. It appears thus important to include nonlinear interactions. In fact, seeing that other differences are found using the real cardiorespiratory data and the linear surrogates, it might be interesting for future studies to consider both computations, as information on linear and nonlinear interactions can be deduced separately.

### Methodological Comments and Future Work

In cardiorespiratory time series, information dynamics are typically determined using a beat-to-beat approach, and thus not by resampling of the time series. However, taking into account that there are substantial differences between the heart rates during rest (typically around 60 bpm) and stress (up to 120 bpm), we chose to resample the tachogram such that lags up to 5 s can be taken into account in the conditioning vector.

A last remark concerns the need for stationary data segments to assess the information dynamics. For this purpose, the data were high-pass filtered and the transient behavior at the start of a mental task was discarded by excluding the first minute of each task. Additionally, ordinal sampling was used for the cardiorespiratory time series and their stationarity was tested, resulting in exclusion of data of 5 subjects due to nonstationary behavior. However, time-frequency analyses showed that the strongest effect of mental stress occurs in the first two minutes of the task [26]. Therefore, there is need for a time-varying approach to capture the information dynamics in fastly varying physiological conditions.

## Conclusion

The aim of this paper was to conduct a combined cardiorespiratory analysis during mental arithmetic and sustained attention via information dynamics. The results suggest that many nonlinearities are present in the cardiorespiratory time series. Also, the comparison between the linear and nonlinear analysis revealed other differences between mental states, motivating not only the use of the nonlinear model-free approach for future studies, but also the approach using the linear surrogates. Additionally, it was shown that, in contrast to the hypothesized reduction in cardiorespiratory coupling, the information transfer and cross information from respiration to HRV were not influenced by the mental state. Likewise, the information storage of HRV seemed to substantially incorporate possible influences from respiration, thus exhibiting no differences between mental states. On the other hand, the internal information measured by the conditional self-entropy of HRV given respiration showed higher predictability during mental stress compared to sustained attention or rest, thus appearing as a very informative quantity for reflecting different levels of activation of the sympathetic nervous system evoked by graded levels of stress.
